# Properties of S-Functionalized Nitrogen-Based MXene (Ti_2_NS_2_) as a Hosting Material for Lithium-Sulfur Batteries

**DOI:** 10.3390/nano11102478

**Published:** 2021-09-23

**Authors:** Chenghao Yao, Wei Li, Kang Duan, Chen Zhu, Jinze Li, Qingyin Ren, Gang Bai

**Affiliations:** 1 College of Electronic and Optical Engineering and College of Microelectronics, Jiangsu Optical Communication Engineering Technology Research Center, Nanjing University of Posts and Telecommunications, Nanjing 210023, China; 1219023526@njupt.edu.cn (C.Y.); 1219023527@njupt.edu.cn (K.D.); Zhuchen@njupt.edu.cn (C.Z.); LiJinze@njupt.edu.cn (J.L.); Rqy@njupt.edu.cn (Q.R.); Baigang@njupt.edu.cn (G.B.); 2 Jiangsu Province Engineering Research Center for Fabrication and Application of Special Optical Fiber Materials and Devices, Nanjing 210093, China; 3 State Key Laboratory of Luminescent Materials and Devices, South China University of Technology, Guangzhou 510641, China; 4 State Key Laboratory of Bioelectronics, Southeast University, Nanjing 210096, China

**Keywords:** Li-S batteries, first-principles study, S-functionalized Ti_2_N

## Abstract

Lithium-sulfur (Li-S) batteries have received extensive attention due to their high theoretical specific capacity and theoretical energy density. However, their commercialization is hindered by the shuttle effect caused by the dissolution of lithium polysulfide. To solve this problem, a method is proposed to improve the performance of Li-S batteries using Ti_2_N(Ti_2_NS_2_) with S-functional groups as the sulfur cathode host material. The calculation results show that due to the mutual attraction between Li and S atoms, Ti_2_NS_2_ has the moderate adsorption energies for Li_2_S_x_ species, which is more advantageous than Ti_2_NO_2_ and can effectively inhibit the shuttle effect. Therefore, Ti_2_NS_2_ is a potential cathode host material, which is helpful to improve the performance of Li-S batteries. This work provides a reference for the design of high-performance sulfur cathode materials.

## 1. Introduction

**Figure 1:**
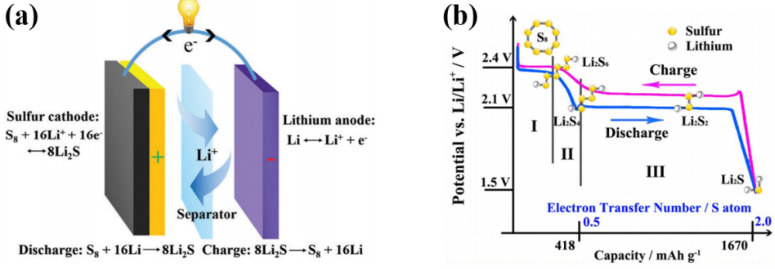
(**a**) Schematic of the electrochemistry, reprinted from [3]. Copyright 2016 with permission from Royal Society of Chemistry. (**b**) Charge-discharge profiles of Li-S batteries, reprinted from [4]. Copyright 2016 with permission from Elsevier.

Presently, the continuous development of electric vehicles and electronic devices puts more requirements on rechargeable batteries [1]. At present, the technology of lithium batteries is relatively mature, but the low theoretical capacity of them cannot meet the needs of future development [2]. Therefore, new rechargeable battery technologies need to be developed. In the next generation of rechargeable batteries, Li-S batteries have received widespread attention because of their high theoretical specific capacity and high energy density. The charge and discharge of Li-S batteries are based on a chemical reaction: S_8_ + 8Li_2_ ↔ 8Li_2_S. During discharging process, the lithium anode is oxidized to form lithium ions and electrons. The lithium ions and electrons travel to the cathode via a membrane and an external circuit, respectively. Sulfur is reduced at the cathode and reacts with lithium ions and electrons to first form soluble intermediates Li_2_S_8_, Li_2_S_6_, Li_2_S_4_, and then form insoluble Li_2_S_2_ and Li_2_S (Figure 1). The charging process is reversed [3,4]. The theoretical specific capacity of Li-S batteries can reach 1675 mAh·g^−1^, and the theoretical energy density can reach 2600 Wh·kg^−1^ [5,6,7]. In addition, as a cathode material, the sulfur has the advantages of large storage capacity, low cost, environmental friendliness, and non-toxicity [8,9,10]. However, in the process of charging and discharging, the long-chain soluble polysulfides (Li_2_S_8_, Li_2_S_6_, Li_2_S_4_) produced at the cathode of Li-S batteries are easily dissolved in the electrolyte. These soluble lithium polysulfides (LiPSs) can shuttle with the electrolyte to the anode (“shuttle effect”), causing the loss of cathode active materials. As a result, the coulombic efficiency of the Li-S batteries is reduced, and the cycle stability is deteriorated [11,12,13]. In addition, sulfur and its discharge products Li_2_S/Li_2_S_2_ have poor conductivity [14]. The application of Li-S batteries is hindered by these problems.

To solve the above-mentioned problems, a lot of efforts have been made. Physical confinement is one of the effective methods [15]. Various structures, such as the open porous structure [16] and the lithium permeable shell [17], have been shown to inhibit the shuttle effect. In 2009, Nazar et al. [18] used CMK-3, a mesoporous carbon, as a conductive skeleton loaded with elemental sulfur, greatly improving the performance of the cathode. In 2018, Ma [19] et al. used the hollow carbon sphere structure as the main body of the sulfur cathode, which effectively improved the stability of the lithium–sulfur batteries.

Another effective method is chemical binding, which uses host materials with high conductivity and appropriate affinity to capture LiPSs [20]. Therefore, a variety of materials have been introduced into sulfur cathodes as host materials, such as graphene [21,22,23], two-dimensional transition metal sulfides and oxides [24,25,26], phosphorene [27,28,29], etc., which have been proved to be possible as cathode host materials.

Recently, *MXenes*, a new type of two-dimensional materials, have received extensive attention due to their high specific surface area, good electrical conductivity and stable structure [30,31,32]. It is considered to have great potential to become excellent sulfur cathode host materials. In 2015, Xiao Liang et al. [33] introduced Ti_2_C into the cathode of Li-S batteries and produced the 70 wt.% S/Ti_2_C composite materials, which proved that there was a strong interaction between LiPSs and Ti atoms on the surface of Ti_2_C. This allowed the specific capacity of the sulfur cathodes to reach 1200 mAh·g^−1^, therefore improving the cycle performance of the sulfur cathodes. The sulfur cathodes still had a capacity retention rate of 80% after 400 cycles of charging and discharging at a rate of 0.5 C. In 2018, Chang Du et al. [34] used Ti_2_O hollow nanospheres to wrap sulfur, which was then embedded in the Ti_2_C interlayer to produce the S@Ti_2_O/Ti_2_C composite materials as the cathodes of Li-S batteries. When the S@Ti_2_O/Ti_2_C composite cathode was charged and discharged at a rate of 0.2 C, its initial capacitance reached 1408.6 mAh·g^−1^. Under the conditions of 2 C and 5 C high-rate charging and discharging after 200 cycles, it could maintain the specific capacities of 464.0 mAh·g^−1^ and 227.3 mAh·g^−1^, respectively.

As *MXenes* are etched with HF acid, functional groups are inevitably left on the surface of *MXenes* [35]. Common natural functional groups are –O, –F, –OH [36]. The presence of functional groups affects the anchoring effects of *MXenes* on LiPSs. In 2019, Dashuai Wang et al. [37] studied the anchoring effects of Ti_3_C_2_ surface functional groups on LiPSs through first-principles calculations. The results showed that the anchoring effects of O-functionalized Ti_3_C_2_ (Ti_3_C_2_O_2_) on LiPSs were better than those of F-functionalized Ti_3_C_2_ (Ti_3_C_2_F_2_). Recently, some studies have shown that it is possible to introduce non-natural functional groups, such as S-functional groups, through experimental means. Unlike natural functional groups, there are few studies on non-natural functional groups. In this work, through first-principles calculations, the adsorption capacity, electronic properties and catalytic capacity of S-functionalized Ti_2_N (Ti_2_NS_2_) for LiPSs are studied. The research results show that Ti_2_NS_2_ has a moderate adsorption capacity for LiPSs, which is stronger than O-functionalized Ti_2_N (Ti_2_NO_2_). In addition, Ti_2_NS_2_ has good electrical conductivity, and it still has good electrical conductivity after adsorption of Li_2_S_x_ species. Therefore, Ti_2_NS_2_ has the potential to become host materials for the cathodes of Li-S batteries.

## 2. Method and Computational Details

In this work, all first-principles calculations are based on the CASTEP package. The exchange-correlation functional is described by the Perdew-Burke-Ernzerhof (PBE) functional within generalized gradient approximation (GGA) [38]. The Grimme of DFT-D2 is used to describe the van der Waals (*vdW*) interaction between the substrate and LiPSs [39,40]. The models of *MXenes* are constructed using 3 × 3 super cells. The size of the vacuum layer is set to 20 Å along the Z-axis to avoid layer-to-layer interaction. To ensure the accuracy of the calculation, 520 eV is selected as the cut-off energy of the plane wave base. The 6 × 6 × 1 k-point grid is used for structural optimization, and the 9 × 9 × 1 k-point grid is used for the calculation of the density of states. Meanwhile, the maximum values of the energy standard, force standard position and displacement standard for structural convergence are 2 × 10^−5^ eV/atom^−1^, 0.05 eV/Å^−1^ and 0.002 Å, respectively. The electron transfer is calculated using the Hirshfeld population analysis method.

The adsorption energy (*E_ads_*) between Li_2_S_x_ species and *MXenes* is defined by the following formula:

(1) $$E_{ads}=E_{species+MXene}-(E_{species}+E_{MXene})$$

where *E_species+MXene_* represents the energy of the entire system after *MXenes* adsorb Li_2_S_x_ species, while *E_MXene_* and *E_species_* represent the energy of isolated *MXenes* and Li_2_S_x_ species, respectively. By definition, the more negative the value, the stronger ability of *MXenes* to adsorb Li_2_S_x_ species.

## 3. Results and Discussion

### 3.1. Structure and Adsorption Performance

**Figure 2:**
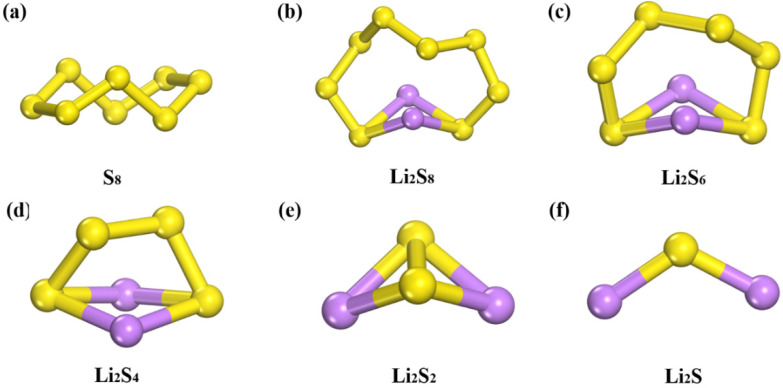
The structures of (**a**) S_8_, (**b**) Li_2_S_8_, (**c**) Li_2_S_6_, (**d**) Li_2_S_4_, (**e**) Li_2_S_2_ and (**f**) Li_2_S. Purple balls represent Li atoms. Yellow balls represent S atoms.

First, the structures of Li_2_S_x_ species are studied (Figure 2). S_8_ presents a folded ring structure, and the shortest S-S bond length is 1.96 Å. The shortest S-Li bond lengths of soluble Li_2_S_8_, Li_2_S_6_, and Li_2_S_4_ are 2.39 Å, 2.41 Å, and 2.37 Å, respectively, and the shortest S-S bond lengths are 2.05 Å, 2.04 Å, and 2.08 Å, respectively. For insoluble Li_2_S_2_ and Li_2_S, the shortest S-Li bond lengths are 2.24 Å and 2.11 Å, respectively. For insoluble Li_2_S_2_ and Li_2_S, the shortest S-Li bond lengths are 2.24 Å and 2.11 Å. All molecules present a 3D structure, not a chain structure, which is consistent with previous work [41].

**Figure 3:**
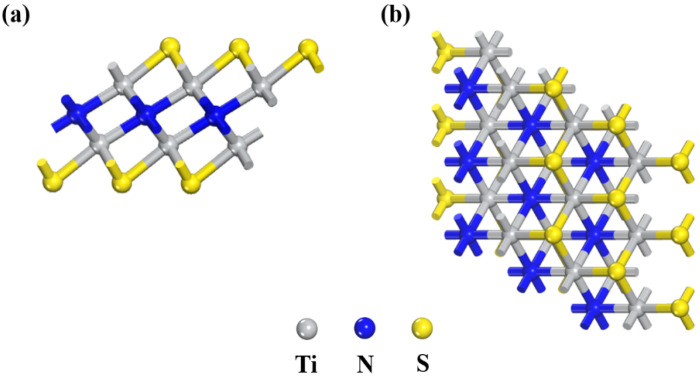
(**a**) Side and (**b**) top views of Ti_2_NS_2_. Yellow balls represent S atoms. Gray balls represent Ti atoms. Blue balls represent N atoms.

Secondly, we establish the model of Ti_2_N (Figure S1). The lattice constant is a = b = 3.01 Å, and the Ti-N bond length is 2.07 Å. Based on Ti_2_N, the model of Ti_2_NS_2_ is established (Figure 3). The fully relaxed Ti_2_NS_2_ presents the hexagonal structure. The lattice constant is a = b = 3.17 Å. The triangular carbon layer in the middle is sandwiched by two triangular titanium layers, while the outermost layer of S atoms is located directly above the lower layer of titanium. Compared with the original Ti_2_N, the Ti–N bond length of Ti_2_NS_2_ changes from 2.07 Å to 2.18 Å. The bond length of the Ti–S bond is 2.39 Å. This is in line with the results of previous research [42], indicating the correctness of the Ti_2_NS_2_ model.

**Figure 4:**
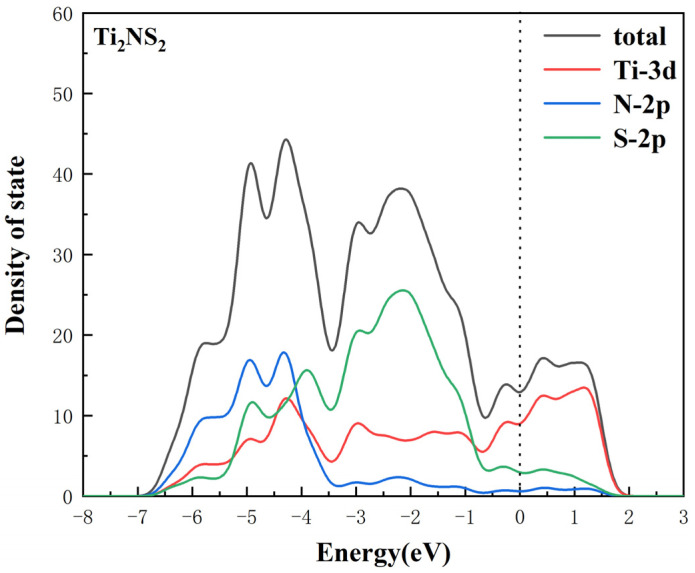
Density of states of Ti_2_NS_2_ (the dotted line indicates the Fermi energy level).

Figure 4 shows the density of states of Ti_2_NS_2_. The dotted line represents the Fermi energy levels. It can be clearly seen from the figure that the Fermi level appears in the electronic state, which indicates that the Ti_2_N with S-functional group presents the metallicity. The metallicity is mainly provided by the d-orbital of titanium. At the same time, the p-orbital of the sulfur atom also contributes to the metallicity of Ti_2_NS_2_. The electrical conductivity of the host materials facilitates the charge-discharge reaction in Li-S batteries, since it can provide the electrons needed for the reaction.

**Figure 5:**
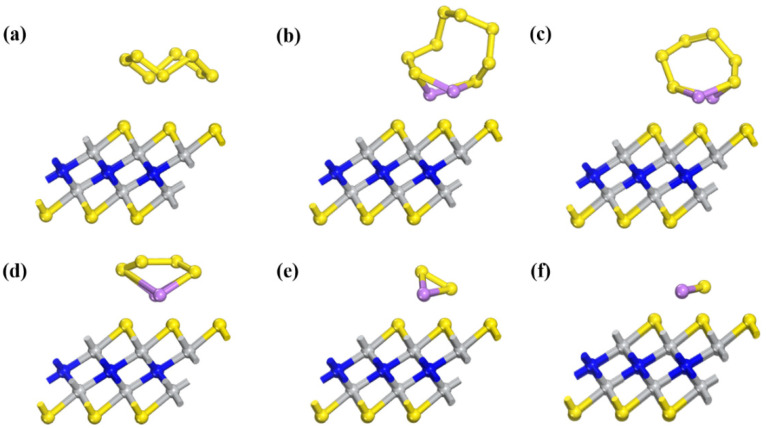
The optimized structures of Ti_2_NS_2_ absorbing (**a**) S_8_, (**b**) Li_2_S_8_, (**c**) Li_2_S_6_, (**d**) Li_2_S_4_, (**e**) Li_2_S_2_, and (**f**) Li_2_S. Purple balls represent Li atoms. Yellow balls represent S atoms. Gray balls represent Ti atoms. Blue balls represent N atoms.

After understanding the structure of Li_2_S_x_ species and Ti_2_NS_2_, the interaction between Li_2_S_x_ species and Ti_2_NS_2_ is studied. To find the stable structures, different positions of the Li_2_S_x_ species on Ti_2_NS_2_ are tried. For Li_2_S, the possible adsorption orientations include S-Top, Li-Side and S-Down (Figure S2). Among the three adsorption orientations, the S-Down becomes the S-Top after optimization, and the adsorption energies of the S-Top and Li-Side are −3.42 eV and −1.56 eV, respectively. Therefore, the S-Top is the most favorable adsorption orientation. The adsorption of Li_2_S_2_, Li_2_S_4_, Li_2_S_6_, Li_2_S_8_ and S_8_ on Ti_2_NS_2_ is considered in a similar manner. The final optimized structures are shown in Figure 5. Table 1 shows the adsorption energies (*E_ads_*), shortest distances between Li_2_S_x_ species and Ti_2_NS_2_ (d), and transfer charge (Q) when Ti_2_NS_2_ adsorbs Li_2_S_x_ species. The ring structure of the S_8_ molecule remains intact, parallel to the surface of Ti_2_NS_2_, and the adsorption energy is −0.57 eV. The shortest distance between the S atom of S_8_ and the S atom on the surface of Ti_2_NS_2_ is 3.52 Å. For insoluble Li_2_S and Li_2_S_2_, their Li atoms tend to combine with the S atoms of Ti_2_NS_2_. Li atoms of Li_2_S and Li_2_S_2_ are surrounded by three S atoms on the surface of Ti_2_NS_2_, and the distances from the nearest S atoms are 2.38 Å and 2.43 Å, respectively, and the adsorption energies are −3.42 eV and −2.36 eV, respectively. As for the soluble Li_2_S_4_, Li_2_S_6_, Li_2_S_8_, their adsorption energies are −1.31 eV, −0.90eV, −0.95 eV, respectively. Similar to the insoluble Li_2_S and Li_2_S_2_, Li atoms tend to combine with the S atoms of the Ti_2_NS_2_, and the shortest distances between them are 2.47 Å, 2.54 Å and 2.51 Å, respectively. Generally speaking, the adsorption energies of Ti_2_NS_2_ for Li_2_S_x_ are between −0.57 eV~−3.42 eV, showing an increasing trend with the deepening of lithiation.

**Figure 6:**
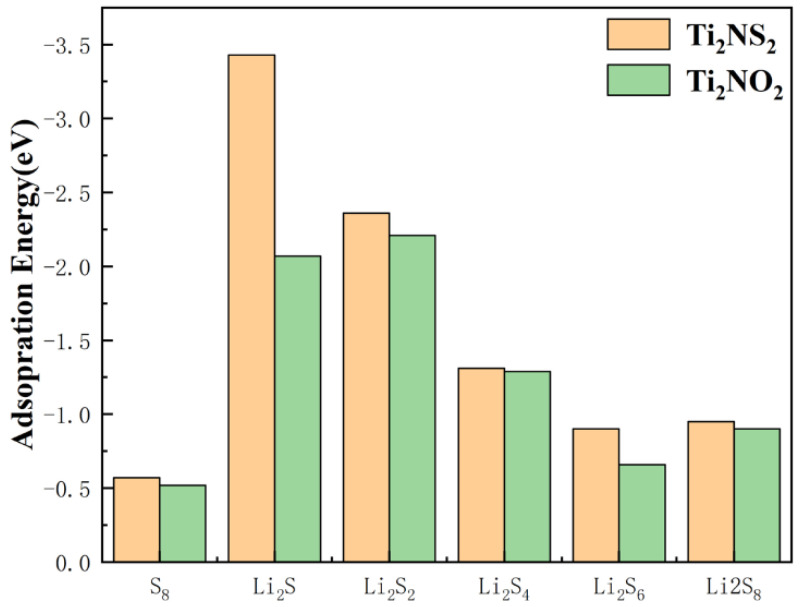
Adsorption energies of Ti_2_NS_2_ and Ti_2_NO_2_.

Since the shuttle effect is caused by the dissolution of soluble polysulfides (Li_2_S_4_, Li_2_S_6_, and Li_2_S_8_) into the electrolyte, we calculate the adsorption energies of electrolyte solvent molecules (DOL and DME) for Li_2_S_4_, Li_2_S_6_, and Li_2_S_8_ (Figure S3). The results show that the adsorption energies of electrolyte solvent molecules are between −0.76~−0.84 eV, which are fewer than those of Ti_2_NS_2_ (−0.90~−1.31 eV). Furthermore, the adsorption energies of Ti_2_NS_2_ are in the range of −0.8~−2.0 eV [43], and the adsorption energy intensity is moderate. Therefore, Ti_2_NS_2_ can effectively inhibit the shuttle effect. In addition, to form a comparison, the model of Ti_2_NO_2_ is constructed (Figure S4). The structure of Ti_2_NO_2_ is similar to that of Ti_2_NS_2_, presenting a hexagon structure. The lattice constant of Ti_2_NO_2_ is a = b = 3.07 Å, and the length of the Ti–O bond is 1.85 Å, which is shorter than that of Ti_2_NS_2_, mainly because the size of the oxygen atom is smaller than that of the sulfur atom. After that, the adsorption energies of Ti_2_NO_2_ for Li_2_S_x_ species are calculated (Figure S5, Figure 6). The results show that the adsorption energies of Ti_2_NO_2_ for Li_2_S_x_ species are −2.07 eV, −2.21 eV, −1.29 eV, −0.66 eV, −0.90 eV, −0.43 eV, respectively, which are fewer than those of Ti_2_NS_2_. Therefore, Ti_2_NO_2_ is less effective than Ti_2_NS_2_ in inhibiting the shuttle effect. The S-functional groups have an advantage over the O-functional groups.

To further explore the adsorption mechanism of Ti_2_NS_2_, the charge transfer and charge density difference between Li_2_S_x_ species and Ti_2_NS_2_ are calculated.

It can be seen from Table 1 that the transferred electrons between S_8_ and Ti_2_NS_2_ are 0.13 e, which indicates that the force between S_8_ and the substrate is weak, and the adsorption energy depends on van der Waals force. Similar to S_8_, the transferred electrons of Li_2_S_8_ and Li_2_S_6_ are 0.15 e and 0.13 e, respectively, so the adsorption energies also mainly depend on van der Waals force. Later, with the deepening of lithium, the transferred electrons become more. The transferred electrons of Li_2_S_4_, Li_2_S_2_ and Li_2_S are 0.22 e, 0.34 e and 0.38 e, respectively. Meanwhile, the adsorption energies become higher, indicating that the transferred electrons affect the adsorption energies.

**Figure 7:**
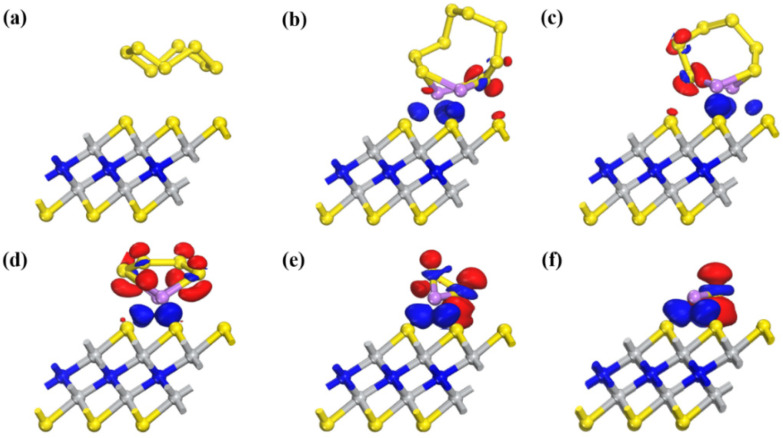
Charge density difference between (**a**) S_8_, (**b**) Li_2_S_8_, (**c**) Li_2_S_6_, (**d**) Li_2_S_4_, (**e**) Li_2_S_2_, (**f**) Li_2_S and Ti_2_NS_2_. The isosurface level is set to 0.025 e/Å^3^. The blue regions indicate charge accumulation, and the red regions indicate charge depletion.

Figure 7 shows the charge density difference between Li_2_S_x_ species and Ti_2_NS_2_. The blue regions indicate the accumulation of charge, and the red regions indicate the depletion of charge. The blue regions are mainly concentrated near the Li atoms of Li_2_S_x_ and the S atoms of Ti_2_NS_2_ surface, which indicates that the transferred electrons between Li_2_S_x_ species and Ti_2_NS_2_ surface are mainly provided by Li atoms of Li_2_S_x_ species. For long-chain sulfides (Li_2_S_8_, Li_2_S_6_, Li_2_S_4_), the blue regions are significantly smaller than those of short-chain sulfides (Li_2_S_2_, Li_2_S), indicating that the transferred electrons of long-chain sulfides are fewer than those of short-chain sulfides, so Ti_2_NS_2_ has a stronger adsorption capacity for short-chain sulfides.

**Figure 8:**
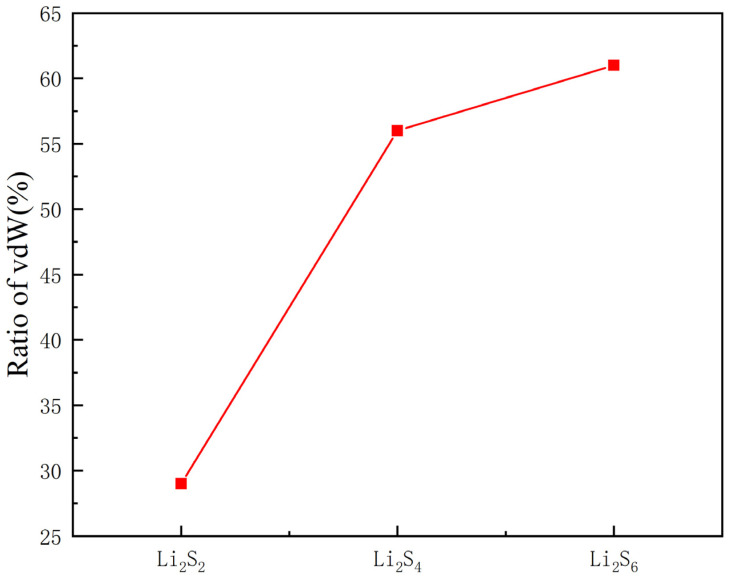
Ratios of *vdW* interaction for Li_2_S_x_ (x = 2, 4, 6) species on Ti_2_NS_2_.

In addition, to better explore the influence of van der Waals forces on adsorption, we take Li_2_S_2,_ Li_2_S_4_ and Li_2_S_6_ as examples to calculate the ratio of *vdW* interaction (*R*), as shown in Figure 8. The R is defined as follows:

(2) $$R=\frac{E_{ads}^{vdW}-E_{ads}^{novdW}}{E_{ads}^{vdW}}\times 100\%$$

where $E_{ads}^{vdW}$ and $E_{ads}^{novdW}$ represent the adsorption energies with and without the *vdW* interaction, respectively. It is clear that the ratio of van der Waals forces decreases and the ratio of chemical interactions increases as the degree of lithium increases. For long-chain sulfides, van der Waals force is the main source of adsorption energy.

### 3.2. Electronic Properties

**Figure 9:**
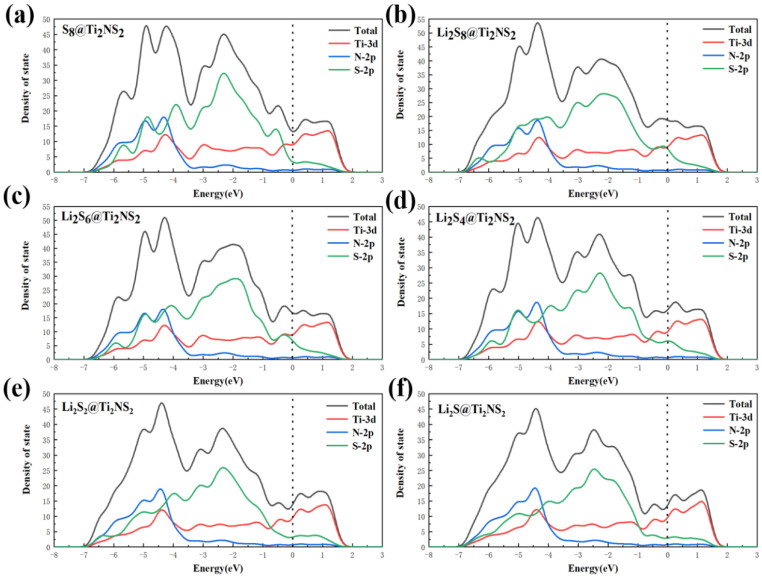
Density of states of (**a**) S_8_, (**b**) Li_2_S_8_, (**c**) Li_2_S_6_, (**d**) Li_2_S_4_, (**e**) Li_2_S_2_ and (**f**) Li_2_S anchored on Ti_2_NS_2_ (The dotted line indicates the Fermi energy level).

It is well known that good conductivity is very important for batteries. However, sulfur, the cathode material of Li-S batteries, is very poor in conductivity. An excellent cathode host material should not only have good conductivity itself, but also have good conductivity after absorbing Li_2_S_x_ species. Therefore, the density of states of the whole systems after Ti_2_NS_2_ adsorbed Li_2_S_x_ species is calculated. Figure 9a shows the density of states of the whole system after Ti_2_NS_2_ adsorbed S_8_, and the dotted line in Figure 9 represents the Fermi energy level. Similar to the density of states of Ti_2_NS_2_, S_8_@Ti_2_Ns_2_ composites still possess metallic properties due to Ti atoms. The electronic properties of S_8_ are changed by Ti_2_NS_2_. In addition, Figure 9b–f show the density of states of the systems which are formed after the adsorption of long-chain sulfides Li_2_S_8_, Li_2_S_6_, Li_2_S_4_ and short-chain sulfides Li_2_S_2_ and Li_2_S by Ti_2_NS_2_. Affected by Ti_2_NS_2_, the composite materials formed by Li_2_S_x_ species and Ti_2_NS_2_ still have an electronic state at the Fermi level. All systems exhibit metallic properties, including S_8_, Li_2_S, and Li_2_S_2_, which are originally poor conductivities. This indicates that the sulfur cathodes can maintain high conductivity during the entire lithiation and delithiation process. This is very beneficial for improving the cycle performance and rate performance of Li-S batteries.

## 4. Conclusions

In this work, the performance of S-functionalized Ti_2_N (Ti_2_NS_2_) as the host materials for the cathodes of Li-S batteries is studied through first-principles calculations. The results show that the adsorption energies of Ti_2_NS_2_ are moderate, stronger than those of Ti_2_NO_2_, especially the adsorption energies of LiPSs are stronger than those of electrolytes, which can effectively inhibit the shuttle effect. At the same time, Ti_2_NS_2_ has good conductivity without adsorption of Li_2_S_x_ species. After adsorption of Li_2_S_x_ species, it still has a high conductivity, which can improve the conductivity of sulfur cathodes and enhance the electrochemical activity during the charge/discharge process. Therefore, Ti_2_NS_2_ has the potential to be the cathode host materials for Li-S batteries. This work provides a reference for the design of high-performance cathode host materials.

## Figures and Tables

**Table 1:** The adsorption energy (*E_ads_*), shortest distance between Li_2_S_x_ species and Ti_2_NS_2_, the charge transfer (Q, a positive value means that the substrate loses electrons from Li_2_S_x_, a negative value is the opposite) when Ti_2_NS_2_ adsorbs Li_2_S_x_ species.

	Li_2_S	Li_2_S_2_	Li_2_S_4_	Li_2_S_6_	Li_2_S_8_	S_8_
*E_ads_*/eV	−3.42	−2.36	−1.31	−0.90	−0.95	−0.57
d/Å	2.38	2.43	2.47	2.54	2.51	3.52
Q/e	0.38	0.34	0.22	0.13	0.15	0.13

## Supplementary Materials

The following are available online at https://www.mdpi.com/article/10.3390/nano11102478/s1. Figure S1: (a) Side and (b) top views of Ti_2_N. Gray balls represent Ti atoms. Blue balls represent N atoms. Figure S2: The possible orientation of Li2S with respect toTi_2_NS_2_. Figure S3: The optimized structures of DME and DOL absorbing (a) S_8_, (b) Li_2_S_8_, (c) Li_2_S_6_, (d) Li_2_S_4_, (e) Li_2_S_2_, and (f) Li_2_S. Figure S4: (a) Side and (b) top views of Ti_2_NS_2_. Red balls represent O atoms. Gray balls represent Ti atoms. Blue balls represent N atoms. Figure S5: The optimized structures of Ti_2_NO_2_ absorbing (a) S_8_, (b) Li_2_S_8_, (c) Li_2_S_6_, (d) Li_2_S_4_, (e) Li_2_S_2_, and (f) Li_2_S. Purple balls represent Li atoms. Red balls represent O atoms. Gray balls represent Ti atoms. Blue balls represent N atoms.
