# Supplementary material for: Properties of S-Functionalized Nitrogen-Based MXene (Ti2NS2) as a Hosting Material for Lithium-Sulfur Batteries
Source: Nanomaterials (Basel). 2021 Sep 23;11(10):2478. doi: 10.3390/nano11102478 (PMC8537390; doi:10.3390/nano11102478)
Supplement: Supplementary file 1 [file nanomaterials-11-02478-s001.zip › nanomaterials-1372684-supplementary.pdf]

Supplementary Materials

Article

Properties of S-Functionalized Nitrogen-Based MXene (Ti_2NS_2) as a Hosting Material for Lithium–Sulfur Batteries

Chenghao Yao [1,4], Wei Li [1,2,3,4,*], Kang Duan [1,4], Chen Zhu [1,4], Jinze Li [1,4], Qingyin Ren [1,4] and Gang Bai [1,4]

1 College of Electronic and Optical Engineering and College of Microelectronics, Jiangsu Optical Communication Engineering Technology Research Center, Nanjing University of Posts and Telecommunications, Nanjing 210023, China; Chenhaoyao@njupt.edu.cn (C.Y.); Kangduan@njupt.edu.cn (K.D.); Chenzhu@njupt.edu.cn (C.Z.); JinzeLi@njupt.edu.cn (J.L.); QingyinRen@njupt.edu.cn (Q.R.); GangBai@njupt.edu.cn (G.B.)

2 State Key Laboratory of Luminescent Materials and Devices, South China University of Technology, Guangzhou 510641, China

3 State Key Laboratory of Bioelectronics, Southeast University, Nanjing 210096, China

4 Jiangsu Province Engineering Research Center for Fabrication and Application of Special Optical Fiber Materials and Devices, Nanjing 210093, China

* Correspondence: liw@njupt.edu.cn

(a) (b)

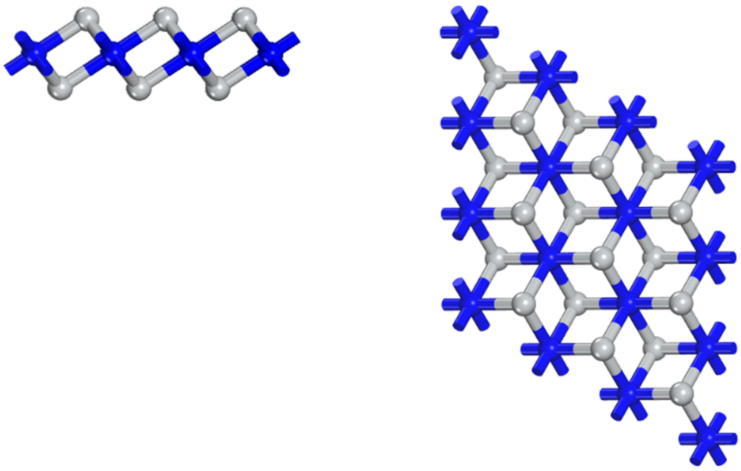

Figure S1. (**a**) Side and (**b**) top views of Ti_2N. Gray balls represent Ti atoms. Blue balls represent N atoms.

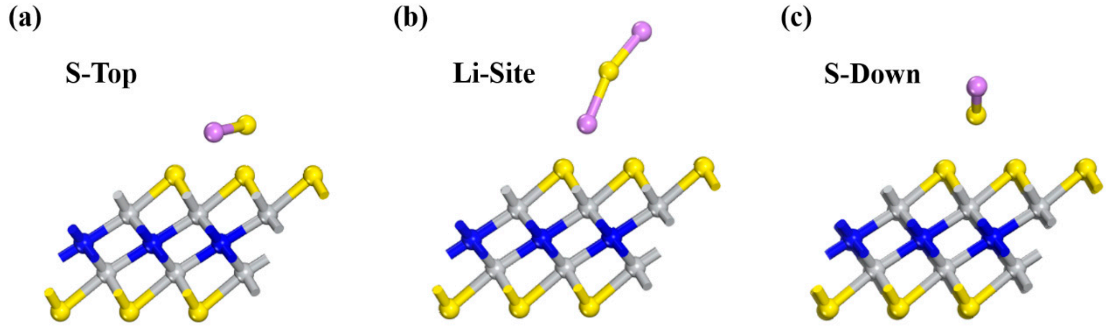

Figure S2. The possible orientation of Li_2S with respect toTi_2NS_2.

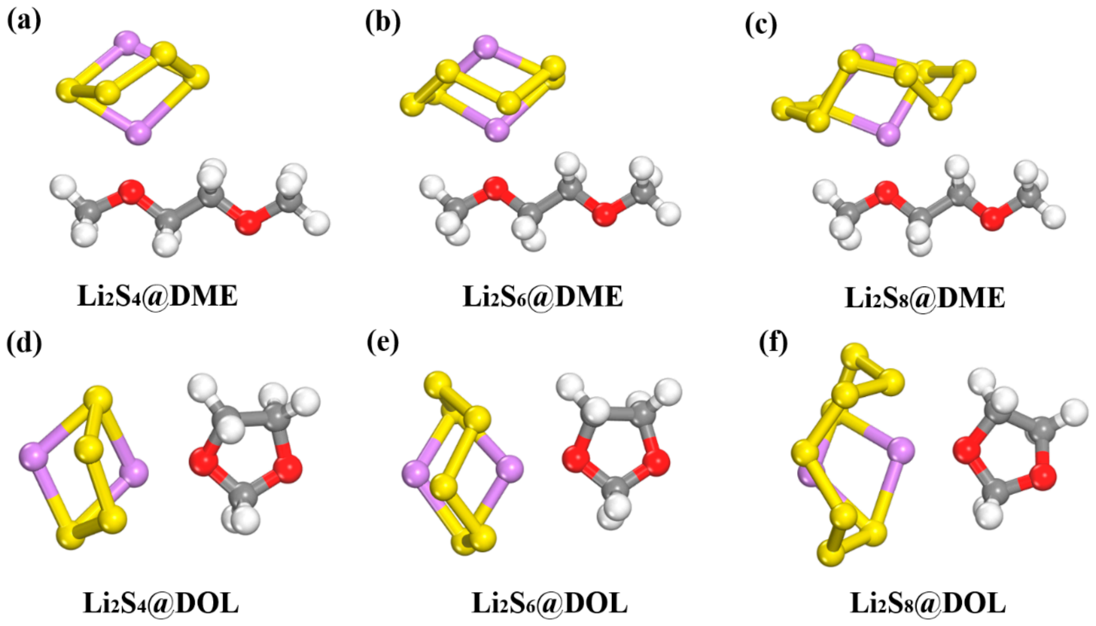

Figure S3. The optimized structures of DME and DOL absorbing (**a**) S_8, (**b**) Li_2S_8, (**c**) Li_2S_6, (**d**) Li_2S_4, (**e**) Li_2S_2, and (**f**) Li_2S.

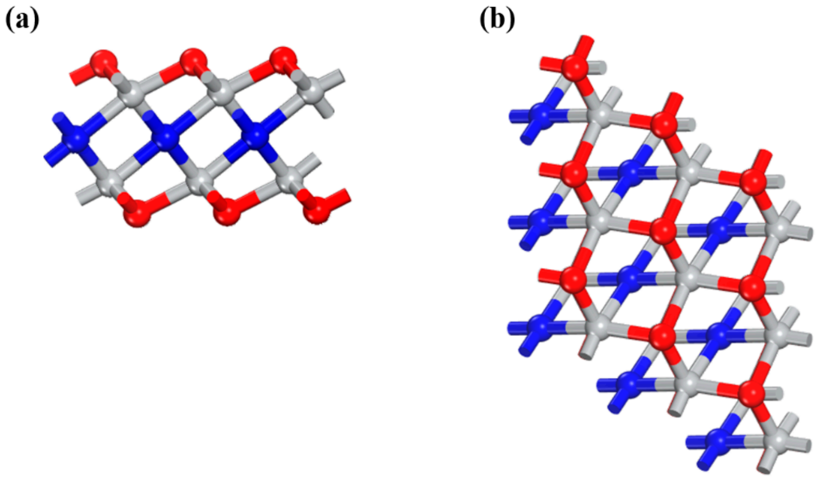

Figure S4. (**a**) Side and (**b**) top views of Ti_2NS_2. Red balls represent O atoms. Gray balls represent Ti atoms. Blue balls represent N atoms.

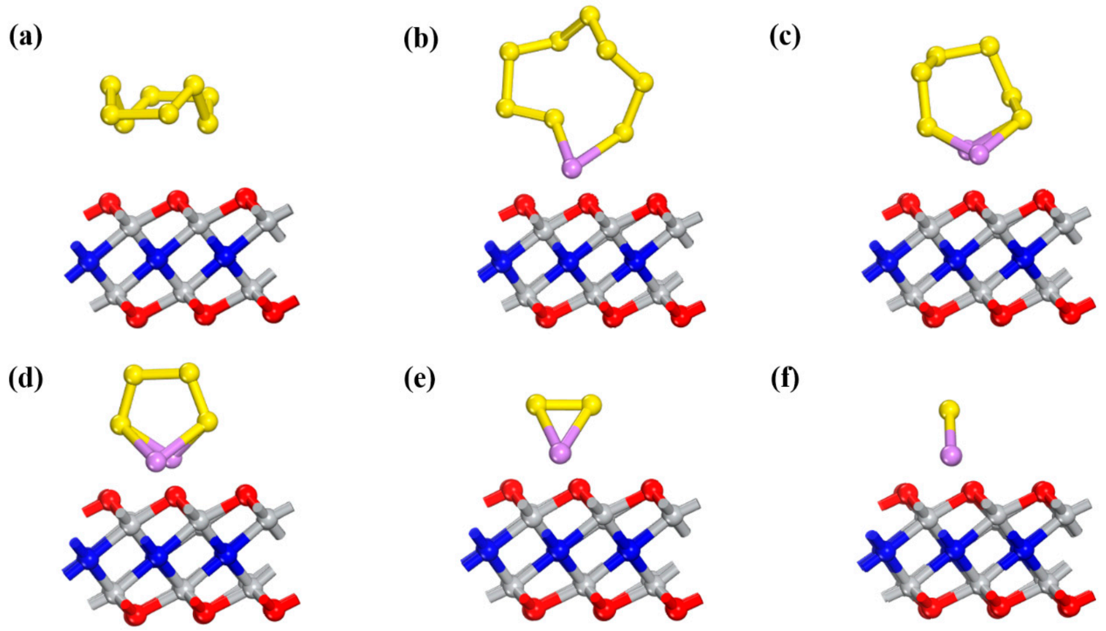

Figure S5. The optimized structures of Ti_2NO_2 absorbing (**a**) S_8, (**b**) Li_2S_8, (**c**) Li_2S_6, (**d**) Li_2S_4, (**e**) Li_2S_2, and (**f**) Li_2S. Purple balls represent Li atoms. Red balls represent O atoms. Gray balls represent Ti atoms. Blue balls represent N atoms.
